# Percutaneous para-levator palpebrae superioris and subconjunctival injection of triamcinolone acetonide for upper eyelid retraction in thyroid-associated ophthalmopathy

**DOI:** 10.3389/fmed.2025.1679057

**Published:** 2025-09-10

**Authors:** Mengdi Wang, Yanan Luan, Zhenbin Qian, Yaohua Wang, Wei Fang

**Affiliations:** ^1^National Clinical Research Center of Ocular Diseases, Eye Hospital, Wenzhou Medical University, Wenzhou, China; ^2^Eye Hospital of Wenzhou Medical University, Hangzhou, China

**Keywords:** thyroid-associated ophthalmopathy, eyelid retraction, triamcinolone acetonide, percutaneous para-levator palpebrae superioris injection, subconjunctival injection

## Abstract

**Background:**

Thyroid-associated ophthalmopathy (TAO) represents the most prevalent inflammatory condition affecting the orbital region. Upper eyelid retraction, predominantly affecting young female patients, can result in notable cosmetic issues and psychological distress. This study aimed to evaluate the therapeutic effectiveness and safety of percutaneous para-levator palpebrae superioris (PLPS) injections compared to subconjunctival (SC) injections via the upper fornix (SC) for the treatment of upper eyelid retraction secondary to TAO.

**Methods:**

This retrospective case–control study encompassed patients with TAO who presented exclusively with upper eyelid retraction. Participants were categorized into either the PLPS group or the SC group based on the injection route. The primary outcome measure was the position of the eyelid margin, quantified by the upper margin reflex distance (MRD1). Secondary outcomes included the incidence of complications and recurrence rates.

**Results:**

The study enrolled 45 eyes from 45 patients, with 24 eyes in the PLPS group and 21 eyes in the SC group. Both groups exhibited significant improvement in upper lid retraction following treatment. In the PLPS group, the mean reduction in MRD1 was 1.67 mm from baseline, with a complete response rate of 45.8%, a partial response rate of 37.5%, and an overall response rate of 83.3%. In contrast, the SC group demonstrated a mean MRD1 reduction of 1.71 mm, with complete and partial response rates of 66.7 and 14.3%, respectively, resulting in an overall response rate of 81.0%. There was no statistically significant difference in therapeutic efficacy between the two groups (*p* = 0.205). However, the incidence of elevated intraocular pressure (IOP) was significantly lower in the PLPS group (1/24 eyes, 4.2%) than in the SC group (6/21 eyes, 28.6%) (*p* = 0.024). No significant differences were observed between the groups concerning menstrual irregularities, post-treatment ptosis, or recurrence rates.

**Conclusion:**

The administration of triamcinolone acetonide (TA) via PLPS injection demonstrates comparable efficacy to SC injection for the treatment of upper lid retraction in TAO, with a reduced incidence of elevated intraocular pressure.

## Introduction

Thyroid-associated ophthalmopathy (TAO) is currently recognized as an autoimmune disorder. It predominantly manifests in patients with Graves’ hyperthyroidism, accounting for approximately 90% of cases, but it may also occur in individuals with Hashimoto’s thyroiditis, in those with thyroid carcinoma, and, albeit rarely, in those with hypothyroidism or euthyroid status (1, 2). The incidence of TAO is markedly higher in women than in men, with annual incidence rates of 16 per million in women compared to 2.9 per million in men (3–5).

Upper eyelid retraction is the most prevalent clinical feature, observed in approximately 58–98% of patients (6, 7). The condition primarily affects individuals aged 20–50 years, with a peak incidence in women in their 30s (1, 8). The asymmetry in facial appearance resulting from eyelid retraction can lead to significant cosmetic concern, particularly among young female patients (9, 10).

The principal pathological mechanism underlying upper eyelid retraction is characterized by inflammatory infiltration and fibrosis of the levator palpebrae superioris (LPS) and Müller’s muscle (11–13). Previous research has indicated that the subconjunctival (SC) administration of triamcinolone acetonide (TA) can effectively target the affected musculature, thereby improving eyelid aperture and appearance (14–23). Nonetheless, the SC route necessitates the eversion of the upper eyelid, which can pose technical challenges in patients who have undergone double-eyelid surgery, potentially resulting in suboptimal TA delivery. Moreover, TA administered via the SC route predominantly impacts Müller’s muscle, with limited efficacy in reaching the LPS. Furthermore, complications such as elevated intraocular pressure (IOP) and glaucoma have been associated with SC injection (15, 24).

Kozaki et al. have previously demonstrated that periocular (percutaneous intra-orbital) injections of TA targeting the region surrounding the LPS can effectively manage inflammation and ameliorate eyelid retraction while minimizing the risk of IOP elevation (25). Consequently, the current study aimed to evaluate the efficacy and safety of percutaneous para-levator palpebrae superioris (PLPS) injections in comparison to SC injections of TA for the treatment of upper eyelid retraction associated with TAO.

## Materials and methods

### Study design and ethical approval

This retrospective case–control study encompassed patients diagnosed with TAO and upper eyelid retraction who received either PLPS or SC TA injections between June 2022 and June 2023 at the outpatient clinic. The study was conducted in accordance with the principles of the Declaration of Helsinki and received approval from the Ethics Committee of the Eye Hospital, Wenzhou Medical University (Approval No.: H2025-031-K-30). Informed written consent was obtained from all participants before their inclusion in the study.

### Inclusion criteria

Patients were included in the study if they met the following conditions: 1. diagnosis of TAO based on Bartley’s criteria; 2. age ≥ 18 years; 3. in primary gaze, the upper lid margin located at or above the corneal limbus (upper margin limbus distance, MLD1 ≥ 0 mm), or an interocular difference in upper lid margin reflex distance (MRD1) ≥ 1 mm; and 4. for bilateral cases, the more severely affected eye was selected for analysis.

### Exclusion criteria

Patients were excluded from the study if they met any of the following conditions: 1. proptosis > 20 mm, strabismus, restrictive ocular motility disorders, or compressive optic neuropathy; 2. use of systemic or local glucocorticoids, immunosuppressants, or biologics within 3 months before treatment; 3. history of botulinum toxin injection within the past 6 months; 4. history of orbital decompression surgery or orbital radiotherapy; 5. presence of ocular myasthenia gravis in the contralateral eye; and 6. IOP > 21 mmHg before the first injection.

### Treatment and grouping

**Figure 1:**
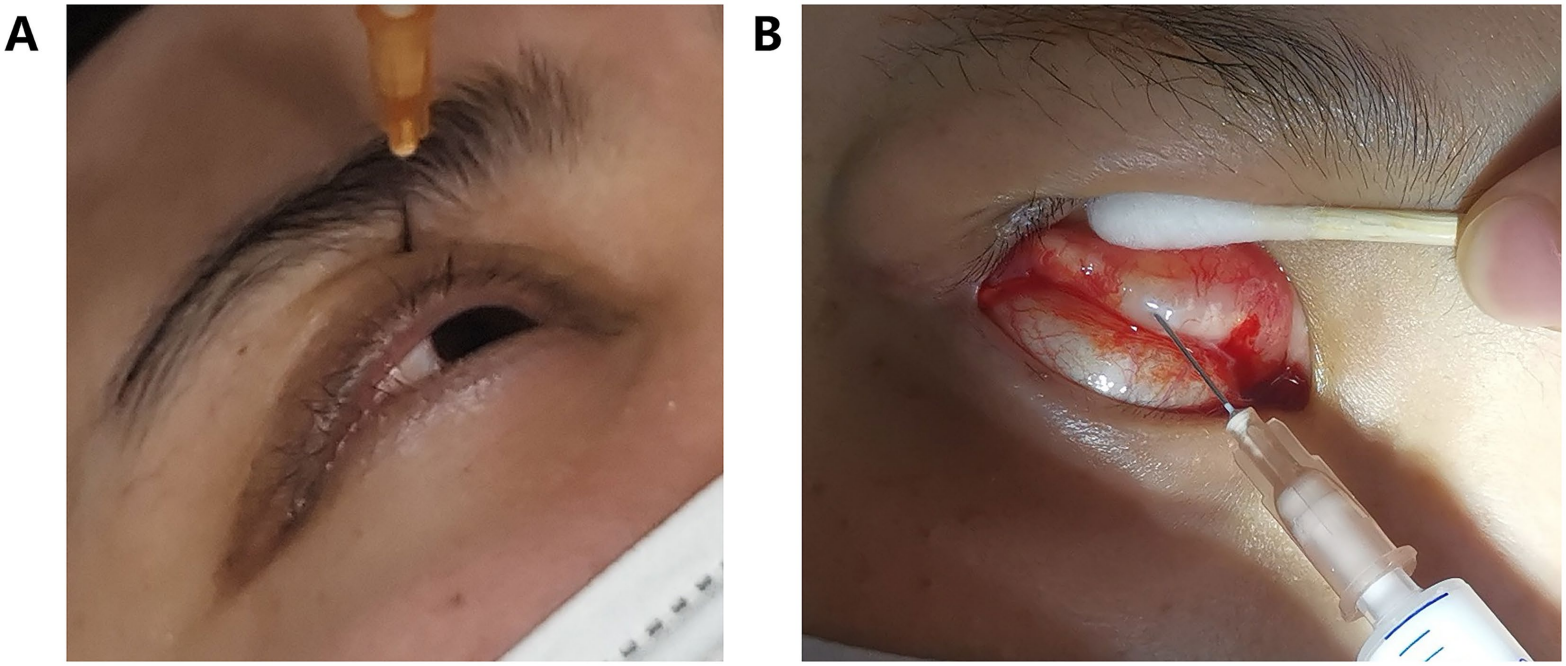
Two triamcinolone acetonide (TA) injection techniques: **(A)** percutaneous para-levator palpebrae superioris (PLPS) injection; **(B)** subconjunctival (SC) injection.

In this study, the therapeutic agent administered was a triamcinolone acetonide (TA) injection (1 mL: 40 mg; Zhejiang Xianju Pharmaceutical Co., Ltd., China). For the PLPS group, a 2-mL syringe equipped with a 25-G needle (38 mm in length) was used. The injection site was identified at the medial one-third of the superior orbital rim, specifically targeting the interspace between the orbital rim and the globe. The needle was inserted perpendicularly along the superior orbital wall to an approximate depth of 1.5 cm, alongside the anterior part of LPS. Before drug administration, aspiration was performed to ensure the absence of blood. A dosage of 40 mg was administered if the MLD1 was greater than or equal to 0 mm, and 20 mg was administered if the MLD1 was less than 0 mm. In the SC group, following the application of topical anesthesia and eyelid eversion, a 1-mL syringe with a 25-G needle (16 mm in length) was employed. The needle was inserted at multiple locations, point by point, approximately 3 mm above the upper edge of the tarsal plate. A total of 0.5 mL (equivalent to 20 mg) of TA was evenly distributed at multiple sites beneath the upper conjunctival fornix. Post-injection, compression was applied to mitigate the risk of bleeding (Figure 1).

### Treatment protocol and study endpoints

1. Injections were administered at intervals of 3 to 4 weeks, with a maximum of six sessions permitted. 2. The treatment endpoint was determined by either patient satisfaction with cosmetic improvement or the absence of significant improvement following three consecutive injections.

### Outcome measures

The primary outcome was the position of the upper eyelid margin, assessed using MRD1 and MLD1. A complete response, or cure, was defined as both upper eyelid margins being located at or below 1 mm above the corneal limbus (MLD1 ≤ −1 mm) and an interocular MRD1 difference of ≤ 0.5 mm. A partial response, deemed effective, was characterized by an MRD1 reduction of ≥ 0.5 mm without meeting the criteria for a complete response. No response, considered ineffective, was defined as an MRD1 reduction of < 0.5 mm. The total response rate was calculated as the sum of the complete response rate and partial response rate.

Secondary outcomes included exophthalmometry measurements and the assessment of complications, such as elevated IOP, ptosis, and menstrual irregularities. Recurrence was defined as a deterioration in eyelid position following 3 months of stability.

### Statistical analysis

Quantitative data were presented as mean ± standard deviation (SD). Categorical data were expressed as counts and percentages. Independent sample *t*-tests were used for normally distributed quantitative variables; the Mann–Whitney U-test was applied for non-normally distributed data. The chi-square test was used for categorical variables. A *p*-value of < 0.05 was considered statistically significant.

## Results

A total of 45 patients (45 eyes) were included in the study. The PLPS group comprised 24 eyes (4 male patients and 20 female patients), with a mean age of 32.13 years, an average of 3.00 injections, and a mean follow-up period of 8.21 months. The SC group consisted of 21 eyes (1 male and 20 female patients) with a mean age of 36.33 years, an average of 3.24 injections, and a mean follow-up period of 7.45 months. No significant differences were revealed between the two groups in terms of gender distribution, age, clinical activity score (CAS), duration of eyelid retraction, thyroid function status, number of injections, follow-up duration, baseline eyelid position, IOP, or proptosis, as detailed in Table 1.

**Table 1:** Comparison of demographic and baseline clinical characteristics between the two groups.

Variable	PLPS group (*n* = 24)	SC group (*n* = 21)	T/U/chi-square value	*p*-value
Gender (men/women)	4/20	1/20	1.607	0.205
Age (years)	32.13 ± 7.48	36.33 ± 7.02	1.938	0.059
Laterality (right/left)	12/12	14/7	1.275	0.259
CAS	0.79 ± 0.72	0.86 ± 0.73	265	0.733
Duration of eyelid retraction (months)	5.48 ± 4.72	8.46 ± 10.89	265.5	0.757
Hyperthyroidism (Yes/No)	18/6	16/5	0.009	0.926
Baseline proptosis (mm)	18.10 ± 2.27	16.98 ± 2.10	−1.72	0.093
Number of injections	3.00 ± 1.52	3.24 ± 1.48	279	0.526
Baseline MRD1 (mm)	5.63 ± 1.14	5.60 ± 1.10	−0.089	0.93
Baseline MLD1 (mm)	−1.02 ± 1.03	0.64 ± 0.98	0.062	0.951
Baseline IOP (mmHg)	14.81 ± 2.58	15.86 ± 2.76	1.317	0.195
Follow-up duration (months)	8.21 ± 7.81	7.45 ± 5.89	256.5	0.918

CAS, clinical activity score; MRD1, upper lid margin reflex distance; MLD1, upper margin limbus distance; IOP, intraocular pressure.

**Figure 2:**
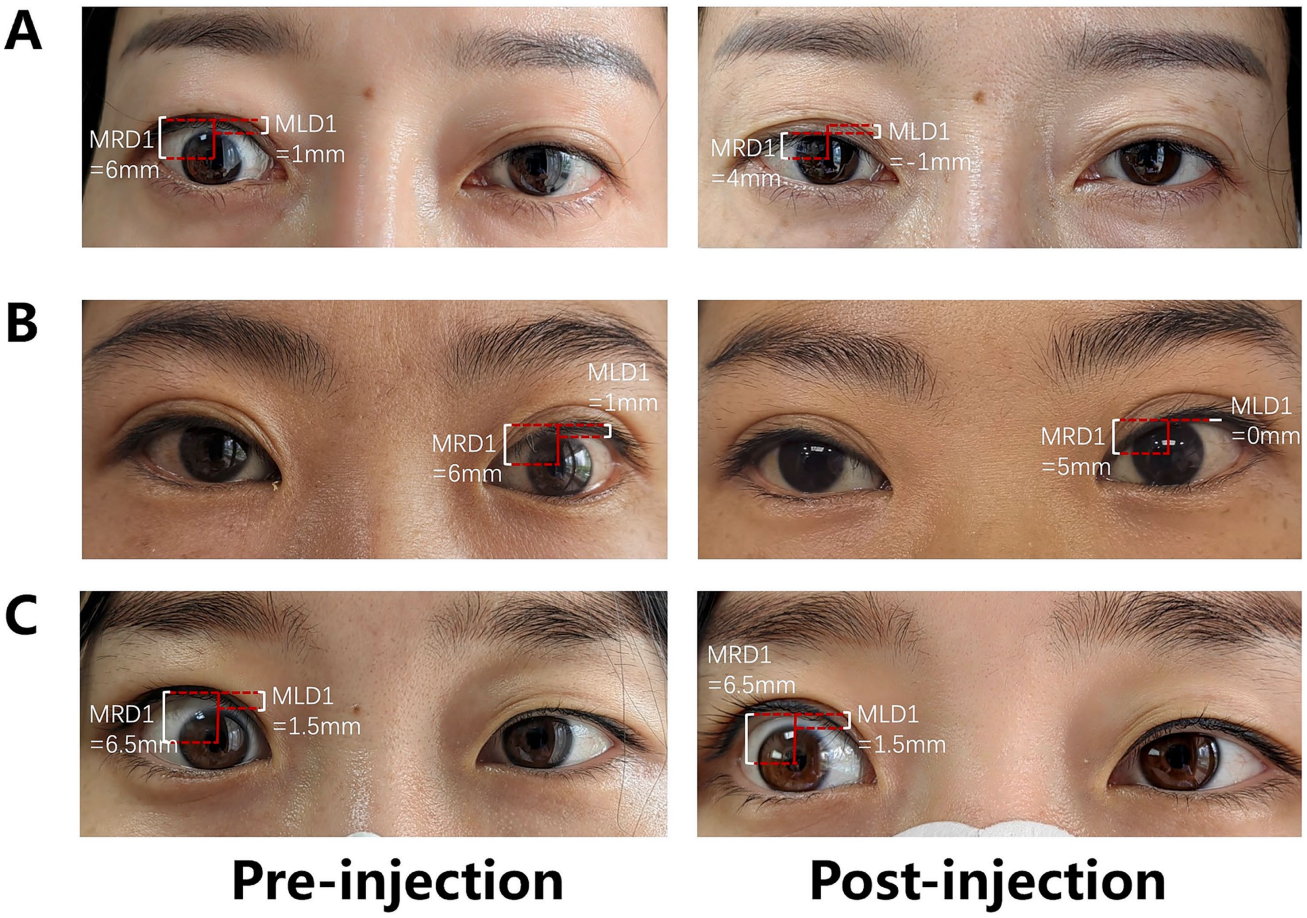
Outcomes following PLPS TA injection. **(A)** Complete response of the right upper eyelid retraction (cured); **(B)** partial response of the left upper eyelid retraction (improved); and **(C)** no response of the right upper eyelid retraction (ineffective).

Following treatment, both groups exhibited significant improvements in upper eyelid retraction. In the PLPS group, the mean MRD1 decreased by 1.67 mm, with a complete response rate of 45.8%, a partial response rate of 37.5%, and an overall response rate of 83.3%. In contrast, the SC group experienced a mean MRD1 reduction of 1.71 mm, achieving a complete response rate of 66.7%, a partial response rate of 14.3%, and an overall response rate of 81.0%. No significant difference was revealed in treatment efficacy between the two groups (*p* = 0.205). Furthermore, comparisons of post-treatment MRD1, post-treatment MLD1, and their respective changes from baseline indicated no significant differences between the groups (*p* = 0.310, 0.624, 0.364, and 0.573, respectively). Additionally, there was no significant difference in post-treatment proptosis between the two groups (*p* = 0.102; Table 2; Figure 2).

**Table 2:** Comparison of treatment outcomes and recurrence between groups.

Outcome	PLPS group (*n* = 24)	SC group (*n* = 21)	T/U/chi-square value	*p*-value
Post-treatment MRD1 (mm)	4.21 ± 1.07	3.88 ± 1.06	−1.027	0.31
ΔMRD1 (mm)	−1.42 ± 1.14	−1.71 ± 1.02	−0.918	0.364
Post-treatment MLD1 (mm)	−0.85 ± 1.25	−1.02 ± 1.03	−0.493	0.624
ΔMLD1 (mm)	−1.48 ± 1.13	−1.67 ± 1.08	−0.568	0.573
Post-treatment proptosis (mm)	18.02 ± 2.14	17.00 ± 1.93	−1.669	0.102
Treatment outcome (cured/Improved/ineffective)	11/9/4	14/3/4	3.174	0.205
Recurrence (n, %)	1 (4.2%)	3 (14.3%)	1.416	0.234

MRD1, upper lid margin reflex distance; MLD1, upper margin limbus distance. ΔMRD1 = post-treatment MRD1 – baseline MRD1; ΔMLD1 = post-treatment MLD1 – baseline MLD1.

The incidence of steroid-induced ocular hypertension in the PLPS group was 1 of 24 (4.2%), which was significantly lower than that observed in the SC group (6/21, 28.6%, *p* = 0.024; Table 3). All patients who developed elevated IOP ceased TA injections immediately. In the PLPS group, the single case of elevated IOP returned to normal with the administration of one topical anti-glaucoma medication. In the SC group, three cases of elevated IOP occurred following the first injection and three after the second injection. Five patients achieved recovery within 3 months using standard anti-glaucoma medications. However, one patient required a quadruple-drug regimen, yet the IOP remained poorly controlled, leading to glaucomatous visual field damage. This patient ultimately underwent canaloplasty, which successfully normalized the IOP.

**Table 3:** Comparison of complications between groups after treatment.

Complications	PLPS group (*n* = 24)	SC group (*n* = 21)	Chi-square value	*p*-value
Elevated IOP	1 (4.2%)	6 (28.6%)	5.078	0.024*
Menstrual irregularities	10/20 (50.0%)	8/20 (40.0%)	0.404	0.525
Ptosis	1 (4.2%)	1 (4.8%)	0.009	1.000

A relatively high incidence of menstrual irregularities was observed in both cohorts following TA injection, with the PLPS group exhibiting a response rate of 50.0% (10/20) and the SC group demonstrating a response rate of 40.0% (8/20). The difference between the groups was not statistically significant (*p* = 0.525; Table 3). The primary clinical manifestations included prolonged menstrual duration and increased bleeding volume. All cases resolved spontaneously within 3 months following the cessation of the drug. Mild ptosis was noted in one patient from each group (*p* = 1.000). Neither of the two cases necessitated special intervention, and both cases resolved spontaneously within 12 months. No additional ocular or systemic complications were observed in either group throughout the treatment and follow-up period.

## Discussion

This retrospective case–control study revealed that TA injections, delivered through both the PLPS and SC approaches, demonstrated comparable efficacy in the management of upper eyelid retraction associated with TAO, with both techniques achieving an overall effectiveness rate exceeding 80%. Nevertheless, the PLPS method was associated with a reduced incidence of IOP elevation, notably a significantly lower occurrence compared to the SC approach (4.2% versus 28.6%). There was no statistically significant difference in the incidence of menstrual irregularities or ptosis between the two treatment groups.

Various peri-orbital injection techniques for TAO have been documented as both effective and safe (26–30). For example, Kozaki et al. conducted single superior intra-orbital injections in 116 eyes of 102 patients with upper eyelid retraction, reporting a 74% improvement rate in eyelid retraction, with no instances of elevated intraocular pressure (IOP) (31). In comparison, our study using PLPS injections demonstrated a slightly higher effectiveness rate of 81%, potentially due to the multiple-injection protocol employed (mean of 3.24 injections). Similarly, the majority of the participants in our study experienced improvement following the initial injection, while a smaller subset required 2–3 injections to achieve satisfactory results. In another study by Bagheri et al., 17 patients (31 eyes) with active TAO, including cases with eyelid retraction, proptosis, and strabismus, received combined triamcinolone and dexamethasone injections at the superior and inferolateral orbital sites, with 3–4 treatment rounds. The improvement rates for upper and lower eyelid retraction were 100 and 68.2%, respectively, with an IOP elevation incidence of 8.8% (32). The results of this study indicate that increasing the frequency of injections may improve therapeutic efficacy; however, it may also elevate the risk of increased IOP.

SC triamcinolone injections demonstrate comparable efficacy to peri-orbital injections but are associated with a higher likelihood of IOP elevation. In a retrospective analysis conducted by Xu et al., involving 97 patients with TAO upper eyelid retraction (126 eyes) treated with SC injections, the cure rate was 64.9%, the effective rate was 22.7%, and the incidence of IOP elevation was 18.6% (15). All 18 patients who experienced elevated IOP were successfully managed with medication or laser treatment, aligning with our findings. In our study, seven patients developed elevated IOP: six cases were managed with medication, while only one patient (in the SC group) ultimately required canaloplasty to normalize IOP and discontinue medication. The pathophysiology of TA-induced ocular hypertension remains unclear. It is hypothesized that corticosteroids may inhibit the degradation of the extracellular matrix and promote the accumulation of glycosaminoglycans in the trabecular meshwork, thereby increasing resistance to aqueous humor outflow and resulting in elevated IOP (24, 33–35). While the majority of patients typically experience spontaneous recovery following the metabolism of the drug, a minority may necessitate interventions such as laser trabeculoplasty to enhance trabecular function or non-penetrating surgical procedures such as canaloplasty. Compared with peri-orbital injections, SC injections at the superior fornix place the drug in closer proximity to the anterior chamber angle, thereby increasing the likelihood of IOP elevation. Furthermore, depot triamcinolone injections at the superior fornix are more challenging to surgically remove than bulbar subconjunctival injections and pose a higher risk of damaging the Müller muscle or LPS, which may result in iatrogenic ptosis (36). Consequently, PLPS injections may be more appropriate for patients with a known predisposition to steroid-induced ocular hypertension.

In this study, approximately 40–50% of female patients in both cohorts experienced menstrual irregularities, which is consistent with the study by Xu et al., which reported a 32% incidence rate (15). These irregularities predominantly manifested as prolonged menstrual cycles and increased menstrual bleeding, with all cases resolving within 3 months after the cessation of drug administration. Following peri-orbital or subcutaneous injection, systemic absorption of triamcinolone can penetrate the hypothalamic–pituitary–gonadal axis. This may inhibit the pulsatile release of gonadotropin-releasing hormone, decrease the secretion of luteinizing hormone and follicle-stimulating hormone, and thereby cause ovulatory dysfunction and menstrual disturbances. Furthermore, the glucocorticoid activity of triamcinolone may suppress adrenal hormone synthesis and, through negative feedback mechanisms, disrupt the regulation of adrenocorticotropic hormone, indirectly impacting ovarian function (37). Nonetheless, existing literature indicates that these alterations are predominantly reversible and generally do not necessitate specific medical intervention (16, 18, 22, 23).

In both groups, one patient each experienced transient, mild ptosis, which resolved spontaneously within 12 months without the need for specific treatment. This condition is likely attributable to mechanical injury to the LPS or Müller muscle fibers during the injection, resulting in temporary muscle weakness. However, some studies have documented cases of irreversible ptosis, potentially associated with local steroid deposition that leads to fibrosis or degenerative changes in the muscle and surrounding tissues, ultimately resulting in muscle weakening or aponeurotic laxity (38).

There was no significant change in proptosis observed before and after treatment in either group, likely because the study exclusively enrolled patients with isolated upper eyelid retraction. In such instances, the pathological changes are confined to the LPS or Müller muscle, with minimal involvement of orbital fat inflammation and limited impact on extraocular muscles, which accounts for the absence of significant proptosis.

The recurrence rate was observed to be lower in the PLPS group than in the SC group (4.2% vs. 14.3%); however, this difference did not reach statistical significance (*p* = 0.234). The underlying causes of recurrence remain undetermined. Potential contributing factors may include variations in thyroid function, persistently elevated levels of thyroid-stimulating hormone receptor antibodies, or insufficient management of orbital inflammation (15, 39).

This study has several limitations. First, the sample size was relatively small. Although the overall effectiveness rates were comparable between the groups, the cure rate was higher in the SC group than in the PLPS group (66.7% vs. 45.8%, *p* = 0.205), necessitating validation in a larger cohort. Nevertheless, due to the heightened risk of steroid-induced ocular hypertension associated with SC injections, we refrained from further increasing the sample size. Second, this study only evaluated the clinical efficacy of triamcinolone injection for upper eyelid retraction in TAO and did not explore factors related to treatment response or morphological changes in the affected muscles, which could be addressed in future research endeavors.

## Conclusion

PLPS injection of TA demonstrates comparable efficacy to SC injection in treating upper eyelid retraction in TAO, with a lower incidence of elevated intraocular pressure. These findings provide a valuable comparison of the safety and effectiveness of both approaches, thereby informing treatment strategies to improve clinical outcomes and minimize complications.

## Data Availability

The original contributions presented in the study are included in the article/supplementary material; further inquiries can be directed to the corresponding author.

## References

[1] Ai K Rishu I Noriko K Toshine M Yoichi I Toshu I. Proptosis in dysthyroid ophthalmopathy: a case series of 10, 931 Japanese cases. Optom Vis Sci. (2010) 87:5702. doi: 10.1097/OPX.0b013e3181ce5702 20081550

[2] Bahn RS. Graves' Ophthalmopathy. N Engl J Med. (2010) 362:726–38. doi: 10.1056/NEJMra0905750, PMID: 20181974 PMC3902010

[3] Raymond SD Nikoo FA Catherine JH Kelvin C Uzma H Patrick R. Increased generation of fibrocytes in thyroid-associated ophthalmopathy. J Clin Endocrinol Metab. (2009) 95:1614. doi: 10.1210/jc.2009-1614, PMID: 19897675 PMC2805489

[4] Huang X Tang W Shen Y He L Tong F Liu S. The significance of ophthalmological features in diagnosis of thyroid-associated Ophthalmopathy. Biomed Eng Online. (2023) 22:7. doi: 10.1186/s12938-023-01073-3, PMID: 36739403 PMC9898900

[5] Tamhankar MA Raza S Brutsaert E Urdaniz E Vainilovich Y Heyes A. The burden of illness in thyroid eye disease: current state of the evidence. Front Ophthalmol (Lausanne). (2025) 5:1565762. doi: 10.3389/fopht.2025.1565762, PMID: 40370423 PMC12075187

[6] Lee DC Young SM Kim YD Woo KI. Course of upper eyelid retraction in thyroid eye disease. Br J Ophthalmol. (2020) 104:254–9. doi: 10.1136/bjophthalmol-2018-313578, PMID: 31079052

[7] Hamed LM Lessner AM. Fixation duress in the pathogenesis of upper eyelid retraction in thyroid orbitopathy. A prospective study. Ophthalmology. (1994) 101:1608–13. doi: 10.1016/s0161-6420(94)38033-x, PMID: 8090464

[8] Yang M He W. Age and gender influence on clinical manifestations of thyroid-associated Ophthalmopathy: a case series of 2479 Chinese patients. Front Endocrinol (Lausanne). (2024) 15:1434155. doi: 10.3389/fendo.2024.1434155, PMID: 39421533 PMC11483995

[9] Wang Y Sharma A Padnick-Silver L Francis-Sedlak M Holt RJ Foley C. Physician-perceived impact of thyroid eye disease on patient quality of life in the United States. Ophthalmol Ther. (2021) 10:75–87. doi: 10.1007/s40123-020-00318-x, PMID: 33196932 PMC7886952

[10] Wang Y Padnick-Silver L Francis-Sedlak M Holt RJ Foley C Douglas RS. Inflammatory and noninflammatory thyroid eye disease: comparison of disease signs, symptoms, and quality of life in patients in the United States. Endocr Pract. (2022) 28:842–6. doi: 10.1016/j.eprac.2022.06.003, PMID: 35714862

[11] Putterman AM. Surgical treatment of thyroid-related upper eyelid retraction. Graded Muller's muscle excision and levator recession. Ophthalmology. (1981) 88:507–12. doi: 10.1016/s0161-6420(81)34987-2, PMID: 7267025

[12] Cruz AA Ribeiro SF Garcia DM Akaishi PM Pinto CT. Graves upper eyelid retraction. Surv Ophthalmol. (2013) 58:63–76. doi: 10.1016/j.survophthal.2012.02.007, PMID: 23217588

[13] Ben Simon GJ Mansury AM Schwarcz RM Modjtahedi S McCann JD Goldberg RA. Transconjunctival muller muscle recession with levator disinsertion for correction of eyelid retraction associated with thyroid-related orbitopathy. Am J Ophthalmol. (2005) 140:94–9. doi: 10.1016/j.ajo.2005.02.034, PMID: 15939390

[14] Badjrai RA Eldinia LR Anandi L Azhari FO Anggraini E Budihardja BM. Triamcinolone injection in the treatment of lid retraction for thyroid-associated ophthalmopathy: a systematic review. Eur J Ophthalmol. (2024) 35:69–76. doi: 10.1177/11206721241254405, PMID: 38751133

[15] Xu D-D Chen Y Xu H-Y Li H Zhang Z-H Liu Y-H. Long-term effect of triamcinolone Acetonide in the treatment of upper lid retraction with thyroid associated Ophthalmopathy. Int J Ophthalmol. (2018) 11:1290–5. doi: 10.18240/ijo.2018.08.07, PMID: 30140631 PMC6090132

[16] Young SM Kim Y-D Lang SS Woo KI. Transconjunctival triamcinolone injection for upper lid retraction in thyroid eye disease—a new injection method. Ophthalmic Plast Reconstr Surg. (2018) 34:587–93. doi: 10.1097/iop.0000000000001120, PMID: 29672347

[17] Duan M Xu D-D Zhou H-L Fang H-Y Meng W Wang Y-N. Triamcinolone Acetonide injection in the treatment of upper eyelid retraction in graves’ Ophthalmopathy evaluated by 3.0 tesla magnetic resonance imaging. Indian J Ophthalmol. (2022) 70:1736–41. doi: 10.4103/ijo.IJO_2228_21, PMID: 35502063 PMC9332998

[18] Lee JM Lee H Park M Baek S. Subconjunctival injection of triamcinolone for the treatment of upper lid retraction associated with thyroid eye disease. J Craniofac Surg. (2012) 23:1755–8. doi: 10.1097/SCS.0b013e3182646043, PMID: 23147303

[19] Huang Z Xu M Zhang W Song X. Triamcinolone Acetonide and botulinum toxin a for upper eyelid retraction in thyroid-associated Ophthalmopathy. Sci Rep. (2025) 15:9063. doi: 10.1038/s41598-025-89063-4, PMID: 39934153 PMC11814297

[20] Yang J Hu X Li Q Huang RL. Botulinum toxin type a injection for correction of upper eyelid retraction in thyroid-associated Ophthalmopathy. J Craniofac Surg. (2023) 34:e485–8. doi: 10.1097/SCS.0000000000009347 37221639 PMC10292564

[21] Kim Y Lew H. Synergistic therapy for graves' Ophthalmopathy-associated eyelid retraction: steroid, 5-Fu, and botulinum neurotoxin a combination. J Clin Med. (2024) 13:12. doi: 10.3390/jcm13103012, PMID: 38792551 PMC11121829

[22] Luo LH Gao LX Wang W Miao H Ma XM Li DM. Triamcinolone Acetonide deep fornix injection for the treatment of upper eyelid retraction in patients with thyroid-associated Ophthalmopathy. Zhonghua Yan Ke Za Zhi. (2020) 56:524–9. doi: 10.3760/cma.j.cn112142-20191009-00504, PMID: 32842335

[23] Xu D Liu Y Xu H Li H. Repeated triamcinolone Acetonide injection in the treatment of upper-lid retraction in patients with thyroid-associated Ophthalmopathy. Can J Ophthalmol. (2012) 47:34–41. doi: 10.1016/j.jcjo.2011.12.005, PMID: 22333849

[24] Liu K Yi J Xu J Zhong L Su N. Risk of intraocular pressure elevation associated with triamcinolone Acetonide administration via different routes in macular edema: a systematic review and network Meta-analysis of randomized controlled trials. BMC Ophthalmol. (2025) 25:150. doi: 10.1186/s12886-025-03979-z, PMID: 40128688 PMC11934556

[25] Kozaki A Nakamura H Inoue T. Clinical efficacy of transcutaneous triamcinolone Acetonide injection for upper eyelid retraction and swelling in patients with thyroid eye disease. Int Med Case Rep J. (2018) 11:325–31. doi: 10.2147/imcrj.S177671, PMID: 30519121 PMC6233704

[26] Young SM Kim YD Woo KI. Nonsurgical Management of Upper Eyelid Retraction in thyroid eye disease. Taiwan J Ophthalmol. (2024) 14:548–53. doi: 10.4103/tjo.TJO-D-23-00043, PMID: 39803393 PMC11717341

[27] Wang Y Du B Yang M Zhu Y He W. Peribulbar injection of glucocorticoids for thyroid-associated Ophthalmopathy and factors affecting therapeutic effectiveness: a retrospective cohort study of 386 cases. Exp Ther Med. (2020) 20:2031–8. doi: 10.3892/etm.2020.8896, PMID: 32782513 PMC7401219

[28] Li D Sun F. Observations on the efficacy of two methods for the treatment of upper eyelid retraction in thyroid-associated Ophthalmopathy. Biomed Res Int. (2021) 2021:9514279. doi: 10.1155/2021/9514279, PMID: 33791385 PMC7997737

[29] Joos ZP Sullivan TJ. Peri-Levator Palpebrae Superioris triamcinolone injection for the treatment of thyroid eye disease-associated upper eyelid retraction. Clin Experiment Ophthalmol. (2017) 45:651–2. doi: 10.1111/ceo.12939, PMID: 28248444

[30] Parsons SR Wilson-Pogmore A Sullivan TJ. Percutaneous triamcinolone injection for upper eyelid retraction in thyroid eye disease. Front Ophthalmol (Lausanne). (2024) 4:1388197. doi: 10.3389/fopht.2024.1388197, PMID: 38984143 PMC11182225

[31] Kozaki A Inoue R Yaji N Nishiyama K Inoue T. Subcutaneous injections of triamcinolone Acetonide for upper eyelid retraction and swelling associated with thyroid eye disease: a retrospective case series study. Clin Ophthalmol. (2024) 18:2147–54. doi: 10.2147/opth.S456543, PMID: 39070106 PMC11277827

[32] Bagheri A Abbaszadeh M Yazdani S. Intraorbital steroid injection for active thyroid Ophthalmopathy. J Ophthalmic Vision Res. (2020) 15:69–77. doi: 10.18502/jovr.v15i1.5948, PMID: 32095211 PMC7001014

[33] Qu Y. Subconjunctival injections of triamcinolone acetonide to treat uveitic macular edema. Int J Ophthalmol. (2020) 13:1087–91. doi: 10.18240/ijo.2020.07.11, PMID: 32685396 PMC7321941

[34] Tawara A Tou N Kubota T Harada Y Yokota K. Immunohistochemical evaluation of the extracellular matrix in trabecular meshwork in steroid-induced Glaucoma. Graefes Arch Clin Exp Ophthalmol. (2008) 246:1021–8. doi: 10.1007/s00417-008-0800-0, PMID: 18386038

[35] Razeghinejad MR Katz LJ. Steroid-induced iatrogenic Glaucoma. Ophthalmic Res. (2012) 47:66–80. doi: 10.1159/000328630, PMID: 21757964

[36] Kalina PH Erie JC Rosenbaum L. Biochemical quantification of triamcinolone in subconjunctival depots. Arch Ophthalmol. (1995) 113:867–9. doi: 10.1001/archopht.1995.01100070041022, PMID: 7605276

[37] Mens JM Nico de Wolf A Berkhout BJ Stam HJ. Disturbance of the menstrual pattern after local injection with triamcinolone Acetonide. Ann Rheum Dis. (1999) 57:700. doi: 10.1136/ard.57.11.700, PMID: 9924216 PMC1752495

[38] Song A Carter KD Nerad JA Boldt C Folk J. Steroid-induced ptosis: case studies and histopathologic analysis. Eye (Lond). (2007) 22:491–5. doi: 10.1038/sj.eye.6702667, PMID: 17220825

[39] Yamana Y Kashima T Mimura M. Efficacy and systemic effects of a single deep orbital triamcinolone injection for thyroid eye disease. Clin Ophthalmol. (2024) 18:2567–74. doi: 10.2147/opth.S476562, PMID: 39257590 PMC11385927

